# SPRYSEC Effectors: A Versatile Protein-Binding Platform to Disrupt Plant Innate Immunity

**DOI:** 10.3389/fpls.2016.01575

**Published:** 2016-10-20

**Authors:** Amalia Diaz-Granados, Andrei-José Petrescu, Aska Goverse, Geert Smant

**Affiliations:** ^1^Laboratory of Nematology, Wageningen UniversityWageningen, Netherlands; ^2^Department of Bioinformatics and Structural Biochemistry, Institute of Biochemistry of the Romanian AcademyBucharest, Romania

**Keywords:** plant–nematode interactions, *Globodera*, SPRY domain, effectors, SPRYSEC, plant targets

## Abstract

Persistent infections by sedentary plant-parasitic nematodes are a major threat to important food crops all over the world. These roundworms manipulate host plant cell morphology and physiology to establish sophisticated feeding structures. Key modifications to plant cells during their transition into feeding structures are largely attributed to the activity of effectors secreted by the nematodes. The SPRYSEC effectors were initially identified in the potato cyst nematodes *Globodera rostochiensis* and *G. pallida*, and are characterized by a single SPRY domain, a non-catalytic domain present in modular proteins with different functions. The SPRY domain is wide-spread among eukaryotes and thought to be involved in mediating protein–protein interactions. Thus far, the SPRY domain is only reported as a functional domain in effectors of plant-parasitic nematodes, but not of other plant pathogens. SPRYSEC effectors have been implicated in both suppression and activation of plant immunity, but other possible roles in nematode virulence remain undefined. Here, we review the latest reports on the structure, function, and sequence diversity of SPRYSEC effectors, which provide support for a model featuring these effectors as a versatile protein-binding platform for the nematodes to target a wide range of host proteins during parasitism.

## Introduction

Plant-parasitic nematodes are microscopic roundworms that can infect thousands of different plant species, causing severe damage to food crops all over the world (Gheysen and Mitchum, 2011). Annual crop losses due to nematodes amount to $125 billion per year, but this sum may be an underestimate because of improper identification of nematode infestations (Danchin et al., 2013; Jones et al., 2013). Outbreaks of plant-parasitic nematodes have long been controlled by applications of nematicide chemicals to infested soils. However, recent legal bans on the use of most of these highly toxic compounds have sparked a particular interest in biological factors determining the efficacy and durability of different types of nematode resistance in crops.

So far, most of the research on nematode resistance has focused on the obligate biotrophic cyst nematodes (genera *Globodera* and *Heterodera*) and root-knot nematodes (genus *Meloidogyne*) (Jones et al., 2013). In the early stages of an infection, these endoparasites migrate through the roots until they find a suitable plant cell to initiate a permanent feeding site (Gheysen and Mitchum, 2011). Cyst nematodes induce a syncytium, a large assembly of hundreds of adjacent cells joined by partially degraded cell walls. Root-knot nematodes induce multinucleate giant-cells by stimulating a few cells to undergo multiple rounds of mitosis without cytokinesis. The ontogeny of both syncytia and giant cells involves the regulation of hundreds of different plant genes, many of which are related to plant cell growth, differentiation, and defense. The permanent feeding site functions as the sole nutrient source for the nematodes for several weeks. Failure to establish a permanent feeding site results in an arrest of nematode development, in which the nematode is unable to reproduce and the host plant becomes then effectively resistant to infection (Goverse and Smant, 2014).

The massive molecular and cellular changes associated with permanent feeding site establishment in plants are most likely brought about by nematode-secreted effectors (Gheysen and Mitchum, 2011; Quentin et al., 2013). In other fields of plant sciences the formal definition of effector is limited to proteins that suppress plant defense responses (Hogenhout et al., 2009), but for plant–nematode interactions the term is used more broadly. Nematode effectors are defined as proteins and small peptides with a wide range of molecular functions that either assist in host invasion, modulation of plant immune responses, or initiation and maintenance of the permanent feeding site (Mitchum et al., 2013; Quentin et al., 2013). Plant-parasitic nematodes produce effectors mostly in dedicated esophageal glands. Specific subsets of these single-celled organs are active during different stages in the nematode lifecycle. The subventral esophageal gland cells are more active in migratory pre-parasitic and parasitic stages, secreting proteins required for root invasion and nematode movement inside the host. The dorsal esophageal gland cell specializes in secretion during the sedentary stages, most likely producing effectors involved in feeding site formation and maintenance. However, there is no precise functional boundary between the secretions of the subventral and dorsal esophageal glands. The function of some of the effectors, such as suppression of host defense, can extend throughout various stages of parasitism. By contrast, different sets of effectors are released to target specific plant cell processes depending on the stage of the infection. Plant-parasitic nematodes deliver the glandular secretions into the plant through a protractible oral stylet. Although this stylet does not seem to penetrate the plasma membrane of host cells, nematodes are able to deliver effectors both into the apoplast and cytoplasm of recipient cells (Mitchum et al., 2013).

A variety of transcriptome and genome analyses have given insight into the diversity and complexity of the large effector repertoires of root-knot and cyst nematodes (Hewezi and Baum, 2012). As the majority of nematode effectors are novel proteins, only a small subset has been functionally well characterized primarily based on initial sequence homology. For instance, host invasion is mediated by a large panel of plant cell wall modifying proteins with striking similarity to bacterial homologs (Davis et al., 2011; Bohlmann and Sobczak, 2014). Likewise, host cell differentiation during the establishment of the permanent feeding site most likely requires the involvement of nematode effectors with sequence similarity to plant CLE peptides (Mitchum et al., 2012). For novel effectors lacking sequence similarity identifying the molecular target in host cells often provides the first concrete lead toward their biological function [e.g., the effector 19C07 of *Heterodera schachtii* (Lee et al., 2011)]. Besides sequence homology and knowledge of host targets, the level of diversity within effector families has also been used to predict their involvement in plant parasitism [e.g., HYP family from *Globodera pallida* (Eves-van den Akker et al., 2014)]. The rationale for focusing on this sequence diversity is the accelerated evolution, which is typically observed in products of gene families operating at plant–pathogen interfaces. In nematodes, as well as in other plant pathogens, many genes encoding effectors harbor highly polymorphic regions and/or variations in copy number resulting from gene duplications and diversifying selection (Hogenhout et al., 2009; Dodds and Rathjen, 2010).

In this review, we focus on recent reports on the diverse roles of secreted SPRY domain-containing proteins (hereafter named SPRYSEC effectors) in plant-nematode interactions. The SPRYSEC effectors were initially identified in the potato cyst nematodes *G. rostochiensis* and *G. pallida*, the genomes of which show remarkable large expansions of SPRY-domain-containing proteins (Cotton et al., 2014; Mei et al., 2015). While the use of the SPRY domain is widespread among eukaryotes, it mostly occurs in association with other functional protein domains (Perfetto et al., 2013). However, the majority of SPRY-containing proteins in potato cyst nematodes do not harbor other functional domains. In the sections below we describe SPRYSEC effectors as selective modulators of plant defense responses mediated by intracellular immune receptors. Based on currently available data we discuss a model in which the versatility of the SPRY domain as protein binding module enables parasitic nematodes to disrupt diverse host protein complexes required for plant innate immunity.

## Identification of SPRYSEC Effectors in Potato Cyst Nematodes

Before the introduction of new generation sequencing technologies, identifying nematode effectors was a challenging and lengthy process (Davis et al., 2008). In this context, a selective search for nematode proteins that were highly abundant in infective juveniles, were specifically localized to the esophageal glands, and carried a signal peptide for secretion could lead to sound nematode effector candidates.

**FIGURE 1:**
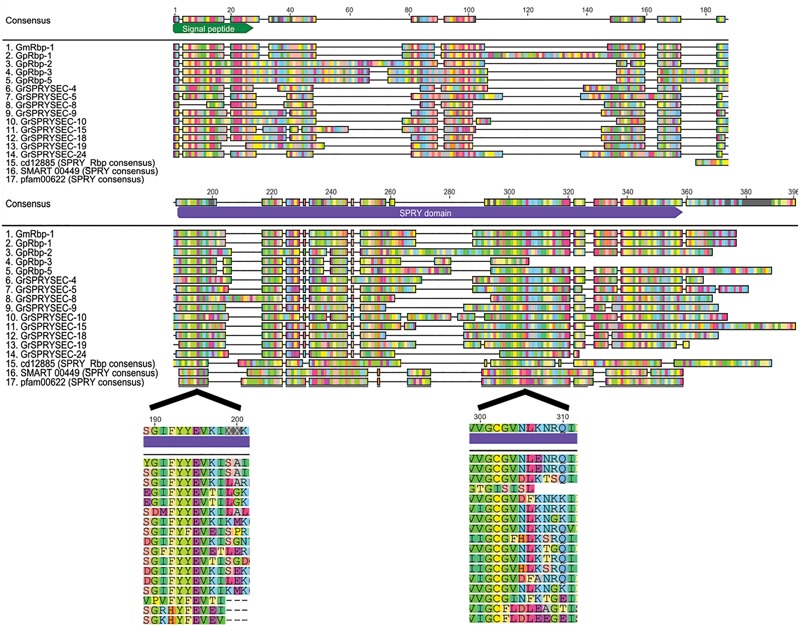
**SPRYSEC effectors are variable in sequence and length.** An alignment of all full-length SPRYSEC effectors available in the NCBI database shows a high degree of sequence variability among them. Sequences starting with Gm are from *Globodera mexicana*, Gp are from *G. pallida* and Gr are from *G. rostochiensis*. The consensus sequences for the SPRY domain from Conserved Domain Database, SMART database and Pfam database are included for reference. The signal peptide and SPRY domain are shown as green and purple blocks in the consensus sequence, respectively. Annotations were done automatically using InterProScan in Geneious 8.1.7 (Quevillon et al., 2005; Kearse et al., 2012). Residues are automatically colored where they are in agreement with the consensus sequence, gray boxes are regions with no agreement with the consensus (Kearse et al., 2012). The enlarged areas show the sequence of the regions where conserved SPRY motifs are found in SPRYSEC effectors.

The application of two differential display approaches using these criteria resulted in the cloning of the first SPRYSEC effectors from *G. rostochiensis* (Qin et al., 2000) and *G. pallida* (Grenier et al., 2002; Blanchard et al., 2005). The genes encoding the SPRYSEC effectors in the two sister species have moderate sequence identity (43.7%) (Blanchard et al., 2005). Further mining of a database with expressed sequence tags of transcripts isolated from (pre-)parasitic juveniles of *G. rostochiensis* resulted in 35 sequence contigs with significant similarity to the original SPRYSEC effector sequences, eight of which contained full length transcripts (Rehman et al., 2009). Recent analyses of the genome sequences of *G. rostochiensis* and *G. pallida* confirmed that the SPRYSEC effectors are members of large, highly diversified gene families (Cotton et al., 2014). The sequence diversity within the SPRYSEC effector families in *G. rostochiensis* and *G. pallida* involves amino acid replacements and significant sequence length variations (**Figure 1**).

The expression of the SPRYSEC genes in potato cyst nematodes specifically localizes to the dorsal esophageal gland cell (Qin et al., 2000; Blanchard et al., 2005; Rehman et al., 2009). Antisera specific to a conserved peptide sequence in the SPRYSEC effectors is also able to detect these effectors in stylet secretions of infective juveniles of *G. rostochiensis* incubated in root diffusates of host plants (Rehman et al., 2009). However, the delivery of the SPRYSEC effectors to either the apoplast or cytoplasm of host cells has not been conclusively shown. This can be partly explained by the fact that the expression and secretion of the SPRYSEC effectors most likely only takes place during the short transition period from migratory to sedentary second stage juveniles (Rehman et al., 2009).

Proteins with a SPRYSEC architecture seem to be rare in nature. The Pfam protein domain database includes around 9000 SPRY domain-containing proteins (PF00622), fifteen percent of which harbor no other functionally annotated domain(s) while about four percent of the latter are predicted to be secreted. Proteins with SPRYSEC architectures are predicted in different eukaryotes, including a number of pathogens and parasites (e.g., the pea aphid *Acyrthosiphon pisum* pfam J9KHA9, *Clavispora lusitaniae* pfam C4Y7R4 and *Entamoeba histolytica* pfam C4M2H6). Because nematode effectors lack sequence similarity to other proteins with SPRYSEC architectures and because no functions have been assigned to other SPRYSEC proteins, it is not clear if the use of a secreted SPRY domain to promote virulence is exclusive to nematodes.

## The SPRY Domain – A Versatile Protein-Binding Platform

The SPRY domain in SPRYSEC effectors was initially characterized as a sequence repeat in tyrosine kinase spore lysis A (splA) from the soil-inhabiting slime mold *Dictyostelium discoideum* as well as in three mammalian ryanodine receptors (Ponting et al., 1997; Rhodes et al., 2005). Concurrently, similar sequence repeats were identified in the product of exon B30.2 in a tripartite motif (TRIM) gene located in the human major histocompatibility complex, which is since then referred to as the B30.2 domain (Vernet et al., 1993). Some aspects of the SPRY and B30.2 domain architectures still remain to be determined with precision. Three sequence motifs (i.e., LDP, YFEVE and LDLE; **Figure 1**) characterize B30.2/SPRY proteins in protein domain databases, with the LDP being absent in the ‘SPRY-only’ group (D’Cruz et al., 2013). The SPRYSEC effectors contain highly conserved variations of the YFEVE (YEVK) and LDLE (VNLK) motifs (**Figure 1**), but not of the LDP motif.

The LDP motif is present in proteins carrying a ∼60 amino acid extension at the N-terminus of the SPRY domain. This extension is cause for debate about the functional boundaries of the domain. In short, the B30.2 configuration is defined by a SPRY domain and an N-terminal extension, the PRY domain (SM00589, PF13765, cl02686), which was initially suggested as a distinct structural element of the B30.2 domain (Rhodes et al., 2005). ‘SPRY-only’ proteins also carry N-terminal extensions of ∼60 amino acids, but these extensions have no significant sequence similarity to the PRY domain. However, the PRY domain and other N-terminal extensions on ‘SPRY-only’ proteins show remarkable similarity in their predicted secondary structure (Woo et al., 2006). Studies with well-characterized members of the ‘SPRY-only’ subfamily show that the N-terminal extension is required for the functionality of the SPRY core domain (Woo et al., 2006). Phylogenetic analyses further suggest that the conserved SPRY core is probably the most ancient part of B30.2/SPRY domain architecture (Woo et al., 2006). The PRY domain and other N-terminal extensions are currently considered an integral part of the B30.2/SPRY domain, albeit more evolutionarily diversified than the core SPRY domain (D’Cruz et al., 2013).

**FIGURE 2:**
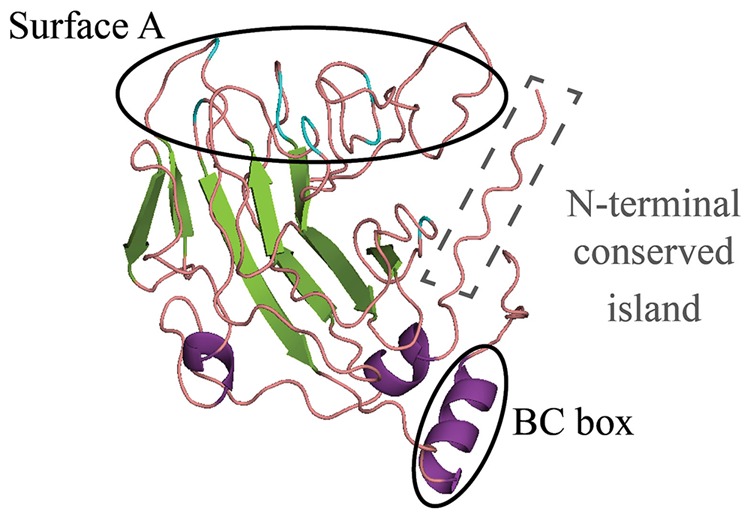
**Remote homology-based structural model of GrSPRYSEC4,5,8,9,15,16,18, and 19 from *G. rostochiensis*.** A remote homology structural model was built for a consensus of these sequences based on the SPRY protein GUSTAVUS (Rehman et al., 2009). The characteristic SPRY β-sheets are shown in green and α-helices in purple. The flexible loops shown in coral and the residues that are found to be under positive selection are colored blue (Rehman et al., 2009). Surface A and BC box, the most hypervariable regions of the characteristic SPRY domain are encircled in black. A conserved island found to be exclusive for nematode SPRYSEC effectors is encircled by a gray dashed line (see also **Figure 3**).

**FIGURE 3:**
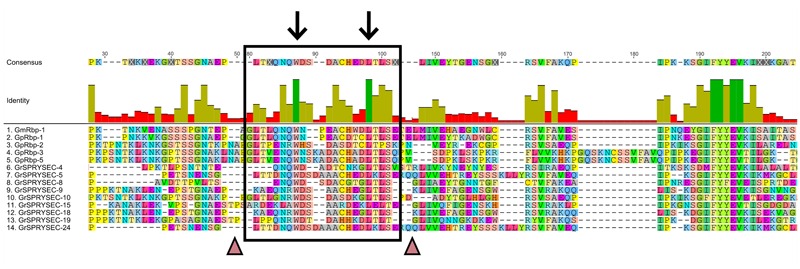
**An N-terminal unique identifier for SPRYSECs.** The N-terminal region of SPRYSEC effectors shows no homology to proteins in the NCBI non-redundant protein database. The black box shows a region with conserved residues in the N-terminus of SPRYSEC effectors. The arrows show 100% conserved positions. The triangles point to areas where insertions of 30–40 residues are usually present depending on the SPRYSEC variant. These insertions have been manually removed for this figure. Colored residues are in agreement with the consensus sequence, gray boxes are regions with no agreement with the consensus. In the identity graph green indicates 100% identity, gold indicates ranges of identity between 30 and 99% and red indicates less than 30% identity (Kearse et al., 2012).

The SPRY domains in SPRYSEC effectors carry an N-terminal extension with lengths varying between 60 and 120 amino acids, depending on the SPRYSEC effector variant. These N-terminal extensions have no significant sequence similarity to the PRY domain or other N-terminal extensions known to be associated with SPRY domains. A PRY domain(s) was initially described in the N-terminus of the SPRYSEC effector GpRbp-1 from *G. pallida* (Blanchard et al., 2005; Carpentier et al., 2012). However, current analyses with domain prediction tools no longer identify a significant match between the N-terminus in GpRbp-1 and PRY domains in domain databases (Pfam, SMART, and CDD). Protein structure modeling of the N-terminal region of GrSPRYSEC-19 from *G. rostochiensis* nonetheless revealed similarities in secondary structure with PRY domain-containing proteins and other “SPRY-only” proteins (**Figure 2**). Furthermore, two highly conserved residues, a tryptophan and a leucine, are found in the N-terminal extensions of all SPRYSEC effectors studied so far (**Figure 3**). Other amino acids in a region of 20 amino acids around these two conserved residues also show high levels of conservation. Protein database searches using only this region suggest that it may be a unique signature sequence of SPRYSEC effectors of nematodes (**Figure 3**).

There is ample evidence showing that the SPRY/B30.2 domain functions as a versatile platform to selectively mediate physical protein–protein interactions (Woo et al., 2006; Perfetto et al., 2013). For instance, the SPRY domain of Ran-binding protein M (RanBPM) mediates interactions required for the activity of RanBPM as a scaffolding protein (Suresh et al., 2012). The SPRY domain in SPRYSEC effectors is most similar to the SPRY domain of RanBPM from various organisms (Blanchard et al., 2005; Rehman et al., 2009). RanBPM carries other domains named LisH, C-terminal to LisH (CTLH) and C-terminal CT11RanBPM (CRA) domains, which are involved in homodimerization (i.e., LisH) and interactions with targets of RanBPM (e.g., the CT11RanBPM domain) (Suresh et al., 2012). However, the SPRY domain in human RanBPM is sufficient to mediate binding of this protein with the transcription factor p73 (Kramer et al., 2004). Similarly, the SPRY domain is also required for binding of human RanBPM to YEPL5, a regulator of the cell cycle progression and cell growth (Hosono et al., 2010). Furthermore, the SPRY/B30.2 domain has undergone a major expansion in the human genome to facilitate the regulation of a wide range of protein–protein interactions in the innate immune system and in antiviral responses [e.g., TRIM proteins; (Perfetto et al., 2013)]. The exact molecular mechanisms underlying the impact of the SPRY/B30.2 domains on other proteins is not well understood, but they often result in alterations in stability of target protein complexes and receptors by ubiquitination and phosphorylation (Perfetto et al., 2013). It is also not known how the peptide-binding specificity is determined in SPRY/B30.2 domains, although it is evident that particular surfaces contribute significantly more to the overall structural diversity in this domain than others (Woo et al., 2006).

## Structural Diversity in SPRY Domains

In crystal structures of SPRY containing proteins the structure of the B30.2/SPRY domain is a compact β-sandwich fold, with two α-helices at the N-terminus (Woo et al., 2006). The β-sandwich is formed by two main layers of β-sheets located in close proximity to each other interacting via a hydrophobic interface. The β-strands are arranged in antiparallel sense and are joined by loops of different lengths that radiate outward from the core sandwich. A structural model of SPRYSEC effectors constructed using as template GUSTAVUS, a SPRY-SOCS box protein from *Drosophila melanogaster*, also predicts a core β-sandwich joined by interspersed flexible loop regions that create exposed surfaces radiating from the β-sandwich core (Rehman et al., 2009).

In the structures of other SPRY-containing proteins highly conserved residues are buried in the core β-sheets of the tertiary structure and therefore are likely required for structural integrity. In comparison, there are no conserved residues in the exposed protein surfaces. This configuration allows the establishment of variable regions in the surface of the SPRY domain that mediate selective protein binding with different targets (Woo et al., 2006). Similarly, mapping of the variable amino acids in SPRYSEC effectors onto a consensus structural model shows that divergent residues mostly localize to the loops that join the core β-sheets of the SPRY domain. The plant targets of SPRYSEC effectors remain largely unknown. However, it is likely that the hypervariable regions formed by the flexible loops of the SPRY domain determine the binding specificity of the SPRYSEC effectors (Rehman et al., 2009). This concept of a stable scaffold with hypervariable regions in extended loops that determine binding specificity for different targets is reminiscent to that of the complementarity determining regions of lectin-binding proteins and immunoglobulins (Masters et al., 2006; Rehman et al., 2009; Perfetto et al., 2013).

In different SPRY-containing proteins the two variable surfaces on the surface of the SPRY/B30.2 domain mediate interactions with other proteins (Woo et al., 2006). This enables SPRY-containing proteins, like SPSB2, to function as E3 ubiquitin ligase, possibly by using one hypervariable region to provide substrate specificity and another to assemble the ubiquitination complex (Kuang et al., 2010). The structural diversity in SPRYSEC effectors is located in multiple predicted exposed hotspots. Thus, a similar model in which a SPRY domain functions as an adapter that joins two host proteins into a complex could apply to SPRYSEC effectors. In a set of SPRYSEC effectors from *G. rostochiensis* the structural diversity concentrates specifically in two surfaces, namely, a hypervariable surface A and a moderately variable alpha helical structure at the C-terminus of the SPRY domain (**Figure 2**; Rehman et al., 2009). The hypervariable regions in the core SPRY domain of SPRYSEC effectors could thus provide substrate specificity to enzymatically active host proteins. For example, SPRYSEC effectors could bind a host target and hijack the cellular machinery of the host to modify their target.

## Genetic Diversity in SPRYSEC Effectors

The relevance of structural diversity in SPRYSEC effectors is also reflected in the large number of gene variants that seem to persist in natural populations of *G. pallida* (Rehman et al., 2009; Sacco et al., 2009; Carpentier et al., 2012). This sequence diversity results from positive diversifying selection, which becomes significant when non-synonymous mutations are favored over synonymous mutations across many generations. Genes participating in the molecular arms race between hosts and parasites typically harbor evidence of positive selection (Jones and Dangl, 2006). For instance, the SPRY domain in TRIM5α proteins that restricts retroviral infections in primates is a hotspot of non-synonymous mutations (Sawyer et al., 2005). Similarly, several amino acid sites mostly located in extended loops that form surface A in the SPRY domain of SPRYSEC effectors in *G. pallida* and *G. rostochiensis* are positively selected (Rehman et al., 2009; Sacco et al., 2009).

The selective forces favoring non-synonymous mutations in SPRYSEC effectors are not fully understood. Changes in amino acid residues that betray the presence of the nematodes to the plant innate immune system can have significant fitness benefits and they seem to contribute to the sequence diversity in SPRYSEC effectors. Position 187 is one of several positively selected sites on the hypervariable surface A of the SPRY domain in GpRbp-1 (Sacco et al., 2009). Multiple variants of the SPRYSEC GpRbp-1 from *G. pallida* carrying a proline at position 187 induce a Gpa2-dependent cell death response in agroinfiltration assays in leaves of *Nicotiana benthamiana* (Sacco et al., 2009). The intracellular NB-LRR immune receptor Gpa2 mediates resistance to specific genotypes of *G. pallida* in potato upon effector recognition (van der Vossen et al., 2000). This characteristic cell death is not observed with nearly identical GpRbp-1 variants carrying a serine at position 187. A single non-synonymous mutation at this position could lead to loss of recognition of *G. pallida* in potato plants harboring Gpa2 resistance. However, cell death-inducing P187 variants of GpRbp-1 seem to persist in populations of *G. pallida* that break Gpa2 resistance and further research is therefore needed to clarify the role of the P-to-S mutation in (a)virulence.

The persistence of cell death-inducing GpRbp-1 variants in nematode populations suggests that these SPRYSEC effectors do not follow a typical birth-and-death scenario. Birth and death scenarios play out when novel positively selected alleles that are not recognized by plant immune receptors become rapidly fixed, resulting in limited overall sequence diversity of pathogen populations (Nei and Rooney, 2005). This particular outcome contrasts with the extensive sequence diversity among SPRYSEC effectors in populations of *G. pallida* (Jones et al., 2009; Carpentier et al., 2012). Non-synonymous mutations in the SPRY domain may therefore also have been instrumental in the functional diversification of the SPRYSEC effectors. In this context, hypervariable sites in the SPRY domain may reflect the ability of the SPRYSEC effectors to function as versatile protein binding platforms to enable interactions with multiple or variable host targets.

The large expansion of the SPRY-domain containing proteins in the genome of *G. pallida* and *G. rostochiensis* also points at extensive functional diversification of the SPRYSEC effectors (Mei et al., 2015; Eves-van den Akker et al., 2016). Gene duplications and recombinations have resulted in approximately 300 SPRY domain-containing proteins in *G. pallida*. Only 30 of these SPRY domain-containing proteins carry a N-terminal signal peptide for secretion and they are therefore considered SPRYSEC effectors. Interestingly, the expression of the SPRYSEC effectors is restricted to the early parasitic stages, while most of the other SPRY-containing proteins are constitutively expressed throughout different life stages. For comparison, Mei et al. (2015) identified far less SPRY domain-containing proteins (<25) in the genomes of *Caenorhabditis elegans, Bursaphelenchus xylophilus*, and *Meloidogyne incognita*, none of which harbors a signal peptide for secretion. The function of the large pool of highly homologous SPRY-domain containing proteins in the genome of *G. pallida* remains to be investigated. However, phylogenetic analysis including most of the 300 SPRY domain-containing proteins in *G. pallida* suggests that they might play an important role in maintaining SPRYSEC effector diversity through intergenic sequence exchanges (Mei et al., 2015).

## SPRYSEC Effectors Suppressing Plant Innate Immunity

Heterologous expression and identification of host targets of SPRYSEC effectors in plants suggest that they may function as suppressors of innate plant immunity. An important line of defense in plants relies on intracellular immune receptors encoded by host specific resistance (*R*) genes that recognize pathogen effectors and activate effector-triggered immunity (Dodds and Rathjen, 2010). Most intracellular plant immune receptors are NB-LRR proteins composed of a central Nucleotide-Binding domain (also known as NB-ARC), and a C-terminal Leucine-Rich Repeat domain. Two major NB-LRR classes are further distinguished based on N-terminal extensions of either a coiled-coil domain (CC-NB-LRR) or a Toll/interleukin 1-like receptor (TIR-NB-LRRs) (Takken and Goverse, 2012). Activation of NB-LRRs upon pathogen recognition commonly leads to defense-related programmed cell death in plant cells. For instance, the resistance mediated by the CC-NB-LRR receptor Mi-1.2 in tomato involves a typical defense-related programmed cell death in the permanent feeding site of the root-knot nematode *M. incognita* (Williamson, 1998).

Five members of the SPRYSEC effector family of *G. rostochiensis* selectively suppress the cell death phenotype triggered by a group of closely related CC-NB-LRRs (Postma et al., 2012; Ali et al., 2015). Remarkably, these SPRYSEC effectors also suppress effector-independent cell death induced by autoactive variants of CC-NB-LRR receptors (Postma et al., 2012; Ali et al., 2015). This suggests that SPRYSEC effectors do not disturb effector recognition by NB-LRR receptors, but rather interfere in downstream signaling. However, the cell death mediated by an autoactive form of NRC1, a downstream signaling component of diverse immune receptors, is not suppressed by the GrSPRYSEC-19 effector (Postma et al., 2012). Furthermore, GrSPRYSEC-19 does not suppress the cell death triggered by elicitin INF1 from the oomycete *Phytophthora infestans*, the onset of which is mediated by an extracellular immune receptor in *N. benthamiana*. By contrast, GrSPRYSEC-19 and several other SPRYSEC effectors of *G. rostochiensis* suppress the cell death induced by the NEP1-like protein PiNPP1.1 of *P. infestans* (Ali et al., 2015). Altogether, these data show that several members of the SPRYSEC effector family in *G. rostochiensis* function as selective suppressors of the defense-related programmed cell death.

At least two SPRYSEC effectors from *G. pallida* (i.e., GpSPRY-12N3 and Gp-SPRY33H17) also selectively suppress the characteristic cell death induced by Gpa2 (Mei et al., 2015). But, unlike SPRYSEC effectors from *G. rostochiensis*, GpSPRY-12N3 and Gp-SPRY33H17 do not suppress Rx1-mediated cell death. GpSPRY-12N3 and Gp-SPRY33H17 do not suppress cell death activated by TIR-NB-LRR-class immune receptors either. Three other members of the SPRYSEC effector family of *G. pallida* (i.e., GpSPRY-17I9-1, GpSPRY-22E10, and GpSPRY-24D4) lack the ability to suppress cell death induced by either Gpa2 or Rx1 in *N. benthamiana* (Mei et al., 2015).

Defense-related programmed cell death is often associated with disease resistance mediated by CC-NB-LRR-class of plant immune receptors, but it is not a requirement for an effective resistance response (Coll et al., 2011). Nevertheless, all of the SPRYSEC effectors of *G. rostochiensis* that suppress cell death in leaves of *N. benthamiana* also suppress resistance to potato virus X mediated by Rx1 (Ali et al., 2015). Co-expression of the resistance gene *N* and the p50 subunit of the *Tobacco mosaic virus* replicase inhibits the accumulation of PVX coat protein fused to GFP (PVX-GFP) in *N. benthamiana* leaves. Co-infiltration of N, p50, PVX-GFP with various SPRYSEC effectors results in enhanced PVX-GFP accumulation in *N. benthamiana* (Ali et al., 2015). Furthermore, stable overexpression of GrSPRYSEC-19 in the diploid potato line V significantly reduced resistance to the wilt fungus *Verticillium dahliae* (Postma et al., 2012).

Host targets of nematode effectors can provide leads to the molecular mechanisms underlying the phenotypes of these effectors in plants. GrSPRYSEC-19 specifically interacts with the C-terminus of the LRR domain alone (Rehman et al., 2009) and with the full-length protein (Postma et al., 2012) of a member of the SW5 *R* gene cluster in tomato (named SW5F). Other members of this cluster of highly conserved CC-NB-LRR proteins are involved in resistance to tomato spotted wilt virus [TSWV; (Spassova et al., 2001)], but none have been linked to nematode resistance in tomato. The function of SW5F in tomato is not resolved, nor is it clear if the SW5F gene encodes a functional protein. Mutations that render other members of the SW5 cluster autoactive, do not result in elicitor-independent SW5F-mediated cell death in *N. benthamiana*. It can therefore not be tested if GrSPRYSEC-19 suppresses the induction of cell death mediated by SW5F in absence of a cognate elicitor. The transient co-expression of GrSPRYSEC-19 with SW5F also does not induce cell death in *N. benthamiana*, which makes it less likely that it is an elicitor of SW5F-mediated cell death and resistance.

## SPRYSEC Effectors Activating Plant Innate Immunity

At least two SPRYSEC effectors trigger a robust cell death response in transient expression assays in leaves of *Nicotiana* species. First, the SPRYSEC effector GpRbp-1 of *G. pallida* induces a Gpa2-dependent cell death in *N. benthamiana* leaves. Conversely, a distant homolog of GpRbp-1 from *G. rostochiensis* does not induce a Gpa2-dependent cell death response, showing that the recognition of GpRbp-1 by Gpa2 is specific (Sacco et al., 2009). Recognition by Gpa2 is also specific within GpRbp-1 variants in the same species. A single amino acid polymorphism S187P in GpRbp-1 abolishes recognition by Gpa2. Gpa2 is known to interact with RanGAP2, a RanGTP-binding protein involved in the nucleocytoplasmic partitioning and functioning of highly homologous immune receptor Rx1 (Sacco et al., 2007). Transient virus-mediated silencing of RanGAP2 in *N. benthamiana* abolishes the cell death mediated by Gpa2 upon recognition of GpRbp-1 (Sacco et al., 2009). Effector recognition and therefore pathogen detection can occur by direct binding to NB-LRRs, however, most examples characterized until now imply indirect recognition of the effector (Dangl et al., 2013). The requirement of RanGAP2 for Gpa2-mediated cell death could indicate that RanGAP2 is monitored by Gpa2 and serves either as a target, decoy, or bait for GpRbp-1 (Sacco et al., 2009). Any of these cases assumes a direct interaction between RanGAP2 and GpRbp-1. While this interaction remains elusive, artificial tethering of RanGAP2 and GpRbp-1 enhances the cell death response mediated by Gpa2 upon detection of GpRbp-1 (Sacco et al., 2009). Introduction of a non-recognized (S187P) variant of GpRbp1 in an artificially tethered complex does not activate Gpa2-dependent cell death. This shows that the interaction with RanGAP2 is therefore involved in recognition of GpRbp-1 by Gpa2 (Sacco et al., 2009).

The second SPRYSEC effector to trigger a cell death response in transient expression assays is SPRYSEC-15 of *G. rostochiensis* (Ali et al., 2015). Unlike the activation of Gpa2-mediated cell death by GpRbp-1, the molecular underpinnings of this cell death response by GrSPRYSEC-15 in non-host *N. tabacum* are not well understood. Heterologous expression of GrSPRYSEC-15 either from a binary expression vector or as a PVX-GrSPRYSEC-15 amplicon induces cell death. Furthermore, expression as PVX-GrSPRYSEC-15 reduces the systemic spread of the virus in *N. tabacum*. Tobacco plants infiltrated with PVX-GFP show chlorotic lesions consistent with systemic spread of the virus. By contrast, plants with PVX-GrSPRYSEC-15 show no symptoms of viral spread 14 days after infiltration. Notably, transient expression of GrSPRYSEC-15 does not induce a cell death response in *N. benthamiana*. These results suggest that an unknown resistance protein in *N. tabacum* most likely recognizes GrSPRYSEC-15, rendering the recombinant PVX-GrSPRYSEC-15 virus avirulent (Ali et al., 2015).

## Perspectives

The SPRY domain in SPRYSEC effectors may provide potato cyst nematodes with a versatile protein-binding platform that allows them to target variable host proteins. In this context, the diversity in SPRYSEC effectors may reflect the variability in the plant targets of these effectors, but on the other hand it may also reflect changes necessary to avoid recognition by the plant immune system. The only consistent plant phenotypes associated with SPRYSEC effectors so far are suppression and activation of CC-NB-LRR-mediated immune responses. The only confirmed host target of a SPRYSEC effector to date is a CC-NB-LRR protein, the role of which in plant innate immunity needs further investigation. Physical associations between SPRYSEC effectors and CC-NB-LRR proteins would fit both in immune activation and suppression models. In fact, these models are not mutually exclusive as immune suppressing SPRYSEC effectors may compete for binding to CC-NB-LRR receptors with immune activating SPRYSEC effectors (Halterman et al., 2010).

The molecular determinants underlying the binding specificity of SPRY domains in SPRYSEC effectors and how binding could lead to a modification of targeted host proteins remain unknown. A single point mutation in a hypervariable surface of a SPRYSEC effector determines if the effector is recognized by the plant immune system (Sacco et al., 2009). The lack of recognition could be due to interference with the interaction between the SPRYSEC effector and the immune receptor. It is not clear if similar mutations in SPRYSEC effectors have also led to gain of function by acquiring novel affinities for other host targets. Resolving the identity of additional host targets of highly similar SPRYSEC effectors may shed light on binding specificity. Although SPRY domains can confer substrate specificity to enzyme complexes [e.g., E3 ubiquitin ligases; (Kuang et al., 2010)], there is no evidence that the SPRY domain alone exhibits intrinsic catalytic activity. Without known intrinsic catalytic activity, the key to understanding the role of SPRYSEC effectors in nematode virulence is to study alterations of plant native complexes brought about by these effectors. SPRYSEC effectors could act as complex inhibitors either by competitive binding to their plant targets [e.g., bacterial effectors AvrRps4 and HopA1; (Bhattacharjee et al., 2011)] or by mediating post-translational modifications of these targets to prevent formation of a stable native complex in the plant [e.g., bacterial effector HopM1; (Nomura et al., 2006)].

Another important question that remains to be addressed is if only potato cyst nematodes exploit the versatility of the SPRY domain to modify host targets. The large expansion of SPRY domain-containing proteins in nematode genomes could be a tell-tale sign to their importance in nematode–plant interactions. At present, it is not possible to assess if similar expansions of the SPRY domain have occurred in related nematode species, given the availability of the genome sequences of only a small number of plant parasitic nematodes. Homologs of SPRYSEC effectors have not been identified in the genome sequence of the root-knot nematodes (Cotton et al., 2014). Several studies using *de novo* transcriptomics suggest that SPRYSEC effectors might nonetheless be common to different cyst nematodes species and might even be present in migratory plant parasitic nematodes. Entries in non-redundant sequence databases imply that the soybean cyst nematode *H. glycines* harbors at least three SPRYSEC effectors (Genbank accessions JQ074058.1, HQ123260.1, JQ074057.1). Similarly, the transcriptomes of the cereal cyst nematode *H. avenea* (Kumar et al., 2014) and migratory endoparasitic lesion nematode *Pratylenchus coffea* (Haegeman et al., 2011) also include sequences closely matching SPRYSEC effectors. When the genome sequences of a wider panel of plant parasitic nematodes become available, it will be possible using comparative genomics to assess if SPRYSEC effectors and their extraordinary expansion are clade specific. Furthermore studying the roles of more ancient SPRYSEC effectors can help to characterize the homology between SPRYSEC effectors and RanBPM. Alternatively, identifying and characterizing functional homologs of RanBPM in plant parasitic nematodes can provide clues to the function of SPRYSEC effectors and their evolution.

## Author Contributions

All authors listed, have made substantial, direct and intellectual contribution to the work, and approved it for publication.

## Conflict of Interest Statement

The authors declare that the research was conducted in the absence of any commercial or financial relationships that could be construed as a potential conflict of interest.
